# Enhancing Flame Retardancy and Smoke Suppression in EPDM Rubber Using Sepiolite-Based Systems

**DOI:** 10.3390/polym16162281

**Published:** 2024-08-12

**Authors:** Jiawang Zheng, Xu Zhang, Dawei Liu, Liwei Zhang, Yuxia Guo, Wei Liu, Shuai Zhao, Lin Li

**Affiliations:** 1 Key Lab of Rubber-Plastics, Ministry of Education/Shandong Provincial Key Lab of Rubber-Plastics, School of Polymer Science and Engineering, Qingdao University of Science and Technology, Qingdao 266042, China; zjw15164181831@163.com (J.Z.); sdbhjebdhehe@163.com (X.Z.); jksbkfbafhb@163.com (W.L.); 2 Rike Chemical Co., Ltd., Weifang 262400, China; liudawei@rikechem.com (D.L.); zlw15698250119@163.com (L.Z.); guoyx1988@126.com (Y.G.)

**Keywords:** sepiolite, flame retardancy, smoke suppression, EPDM rubber, Enteromorpha, graphene

## Abstract

The burning of Ethylene–Propylene–Diene Monomer (EPDM) rubber generates substantial smoke, posing a severe threat to the environment and personal safety. Considering the growing emphasis on safety and environmental protection, conventional non-smoke-suppressing flame retardants no longer satisfy the present application requirements. Consequently, there is an urgent need to develop a novel flame retardant capable of suppressing smoke formation while providing flame retardancy. Sepiolite (SEP), a porous silicate clay mineral abundant in silica and magnesium, exhibits notable advantages in the realm of flame retardancy and smoke suppression. This research focuses on the synthesis of two highly efficient flame-retardant smoke suppression systems, namely AEGS and PEGS, using Enteromorpha (EN), graphene (GE), sepiolite (SEP), ammonium polyphosphate (APP), and/or piperazine pyrophosphate (PPAP). The studied flame-retardant systems were then applied to EPDM rubber and the flame-retardant and smoke suppression abilities of EPDM/AEGS and EPDM/PEGS composites were compared. The findings indicate that the porous structure of sepiolite plays a significant role in reducing smoke emissions for EPDM composites during combustion.

## 1. Introduction

In recent years, global economic development has reached unprecedented levels, providing individuals with a wide array of choices for materials to embellish their living and working spaces. However, this progress has also brought about increasingly complex fire hazards, posing severe threats to both lives and property safety [1]. Currently, numerous fires, particularly major incidents, are primarily or indirectly caused by building decorations in civil structures and the utilization of polymer-like materials for building insulation. These materials often possess low thermal conductivity and unsatisfactory mass-to-strength ratios [2]. A report [3] states that, on average, smoke from burning materials like these is responsible for approximately two-thirds of the deaths in building fires in the United States each year. The level of smoke hazard significantly limits firefighters’ choice of rescue routes during such incidents. Within a mere 30 s, a fire can rapidly spiral out of control, engendering a substantial release of heat and toxic smoke throughout the area [4]. This smoke can irritate the human body, leading to rapid vision loss, making it difficult to keep one’s eyes open and impeding movement along walls, thereby slowing down escape efforts and increasing the mortality rate [5]. Consequently, the inhalation of copious amounts of toxic and harmful smoke during a fire constitutes a significant cause of death [6,7]. Thus, the investigation of the flame-retardant and smoke suppression properties of diverse materials assumes a pivotal role in safeguarding lives, protecting property, and preserving the environment.

Currently, smoke suppression technology is attracting a lot of attention in the field of polymer flame retardants [8]. Efforts to reduce the amount of smoke generated during combustion generally follow one of the following two paths: one is the use of low-smoke polymer materials; the second is the addition of smoke suppressants, so that the amount of smoke generated during combustion decreases. A comprehensive view of the latter is obviously more feasible. Triphenylphosphine and α,α′-dibromo-p-xylene were used to synthesize a phosphorus-containing porous polymer PPOP2, and the flame-retardant smoke suppressant was studied [9]. The smoke suppression effect of this flame retardant on epoxy resin (EP) was investigated. The results confirmed that the total smoke production could be reduced by 39% when the addition amount was 5 wt%. Two melamine salts (MA)_2_WO_4_ and (MA)_2_MoO_4_, as flame-retardant smoke suppressants, were prepared for flexible PVC by means of dehydrated sodium tungstate and dehydrated sodium molybdate [10]. Among them, (MA)_2_MoO_4_ was more effective in terms of flame-retardant smoke suppression. Dethyl N,N-bis(2-hydroxyethyl)aminomethylphosphonate (FRC-6), 1,6-adipic acid (1,6-AA), and 1,6-hexanediol (1,6-HDO) were used to synthesize phosphorus-containing polyester diol (JZP) and finally to prepare an efficient fire-retardant expandable polystyrene insulation material (AF-EPS) [11]. Metal-based smoke suppressants, which mainly include iron compounds [12] (ferrocene Fe(C_5_H_5_)_2_), molybdenum compounds [13] (MoS_2_), nickel compounds [14] (Ni(OH)_2_), magnesium compounds [15] (Mg(OH)_2_), zinc compounds [16] (ZnB), and rare earth compounds [17], etc., have also been extensively studied worldwide. In addition, the unique lamellar structure of graphene gives it a high specific surface area and strong physical adsorption properties, so it is also widely used in flame-retardant and smoke-suppressing applications. A cross-linked supramolecular poly(cyclotriphosphazene) functionalized graphene oxide (FGO) was synthesized and added into polypropylene (PP) to explore the extent of improvement in flame-retardant and smoke suppression properties [18]. Azam Jamsaz [19] et al. found that graphene-based nanomaterials (GNs) such as graphene and graphene oxide improved the thermal stability of PUFs and delayed their ignition [19]. Yan [20] et al. created a series of new montmorillonite polyphosphate (OPEA) flame retardants by grafting cyclic phosphoric acid (PEA) onto organic modified montmorillonite (OMMT) with different contents, and successfully synthesized and prepared a variety of adhesive sheets by mixing them with melamine formaldehyde resin [20]. Sepiolite (SEP) is a naturally occurring, hydrated magnesium silicate mineral that exhibits a clay-like, needle-like fibrous structure, of which the molecular formula is Si_12_O_30_Mg_8_(OH)_4_(H_2_O)_4_·8H_2_O. The crystal structure of sepiolite closely resembles the 2:1 lamellar structure of montmorillonite (MMT). This structure comprises two silica lamellae with a tetrahedral structure, sandwiched by an octahedral lamella structure composed of magnesium oxygen. The lamellae are connected to each other by means of oxygen atoms, creating channels and pores that contain zeolitic water and cations like potassium and calcium [21]. Sepiolite possesses a tubular penetration channel measuring 0.37 nm × 1.06 nm and a theoretical surface area of 900 m^2^/g. The distinctive sandwich structure, channels, and pores contribute to sepiolite’s exceptional adsorption properties, owing to its large specific surface area and pore volume. Consequently, sepiolite can effectively adsorb volatile gases and small particle volatiles, playing a significant role in smoke suppression during polymer combustion. Additionally, sepiolite’s inner layer contains cations that exhibit both ion-exchange and non-halogen flame-retardant properties. The fibrous structure of sepiolite facilitates its dispersion within polymers [22].

Although natural sepiolite has many excellent properties and has great research potential in the field of flame retardancy and smoke suppression, it has poor compatibility with rubber and other matrices as an inorganic clay mineral. When it is directly added to the rubber matrix, it is easy to agglomerate, which affects the flame retardancy of the material. The dispersion of the modified sepiolite in the matrix can be significantly improved, thereby improving the flame-retardant and mechanical properties of the material. As a result, sepiolite holds great research potential in the field of flame retardancy and smoke suppression. Currently, the most commonly employed modification methods for sepiolite include organic modification, ion exchange modification, acid modification, and roasting modification [23]. Among these methods, the use of silane coupling agents to modify the surface of sepiolite is widely adopted in academia. Silane coupling agents possess siloxy groups that exhibit an affinity for inorganic groups, as well as organic functional groups that exhibit an affinity for organic groups. Therefore, when introduced, the silane coupling agents react with the hydroxyl groups (-OH) on the sepiolite surface, resulting in grafting onto the sepiolite structure [24]. Flame retardants called SPPBs were synthesized by grafting flexible polyphosphate ester (PPB) onto the surface of sepiolite (SEP) [25]. They prepared amino transparent flame-retardant coatings by thoroughly mixing the modified sepiolite with melamine formaldehyde resin and applied them to wood materials. The coatings demonstrated a positive effect of SEP on the thermal stability and smoke suppression properties. Quaternary composites were utilized to improve the smoke suppression performance of the PP/IFR (intumescent flame-retardant) matrix [26]. The addition of sepiolite to the PP/IFR matrix significantly enhanced its smoke suppression performance. Wet synthesis was employed to graft zinc hydroxystannate (ZHS) nanoparticles onto the surface of sepiolite, successfully preparing SEPZHS hybrid additives with uniform nanoparticle distribution and organic modification using aminosilanes to enhance compatibility with EPDM [27]. Modified sepiolite with KH550 was studied to improve the thermal stability of PU due to the thermal insulation effect of KH550-Sp [28]. When 3wt% KH550-Sp was added, the initial decomposition temperature increased by about 20 °C, and the residue weight at 550 °C increased by about 5.8%.

**Figure 1:**
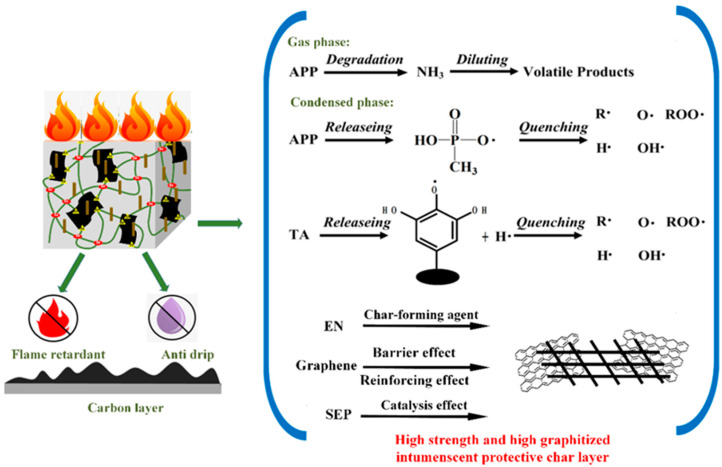
Mode of Action for AEGS and PEGS in EPDM.

In this study, the component tannic acid (TA), Enteromorpha (EN), and sepiolite (SEP) in the studied flame retardants AEGS and PEGS are from nature, which is environmentally friendly. Additionally, the flame retardants AEG and PEG were prepared by compounding with the halogen-free phospho-nitrogen flame retardants ammonium polyphosphate (APP) and piperazine pyrophosphate (PPAP). Consequently, this current study solely focuses on the preparation of AEGS and PEGS composites through the addition of sepiolite (SEP). The flame retardant mechanism is shown in Figure 1. The investigation is focused on the mechanical properties, thermal stability, and flame and smoke suppression properties of EPDM/AEGS and EPDM/PEGS composites.

## 2. Experimental

### 2.1. Materials

EPDM (8550C, containing 5% third monomer) was purchased from Alang Xingke High Performance Elastomer Changzhou Co., Ltd. (Changzhou, China). The average particle size of Enteromorpha (EN) by mechanical grinding treatment (which took place on the southeast coast of Shandong Peninsula in 2017) is 2245 nm; piperazine pyrophosphate (PPAP, JNP-2) was provided by the Sichuan Research Institute of Fine Chemicals; and the silane coupling agent γ-aminopropyltriethoxysilane (KH550) was purchased from Qingdao Hengda Zhongcheng Co. (Qingdao, China). Zinc oxide (ZnO), stearic acid (SA), N-Cyclohexyl-2-benzothiazolinesulfonamide (CZ), 2,2-dibenzothiazole disulfide (DM), sulfur (S), and carbon black (CB) were all industrial-grade and sponsored by Henan Longji Co. (Henan, China).

### 2.2. Modification of Sepiolite

The sepiolite sample was placed in the beaker, and anhydrous ethanol and the silane coupling agent KH550 were added. The mixture was then stirred in a homogenizer for 15 min. The mass ratio of sepiolite to silane coupling agent was 20:1, and the stirring rate was 3000 r/min. Subsequently, the sepiolite modified with KH550 was washed three times with fresh anhydrous ethanol, and then transferred to a blast dryer and dried at 80 °C for 12 h. After the drying process, the modified sepiolite, known as K-SEP, was obtained, weighed, and prepared for further use.

### 2.3. Preparation of AEGS and PEGS Composites

The flame-retardant co-factor EGT was synthesized using graphene (GE), tannic acid (TA), and Enteromorpha (EN). Additionally, the flame retardants AEG and PEG were compounded with ammonium polyphosphate (APP) and piperazine pyrophosphate (PPAP) and K-SEP to prepare AEGS and PEGS [29].

The EPDM compound was prepared by initially mixing EPDM in a 500 mL compactor at 110 °C and 60 rpm/min. Subsequently, zinc oxide, stearic acid, DM, CZ, and carbon black were sequentially added. AEGS and/or PEGS were then compounded with EPDM. Then, the mixture was discharged onto a two-roll mill at 80 °C, where sulfur was added. Immediately after each mixing, the composition was removed from the roll, and while still hot, was passed once through a cold two-roll mill to achieve a sheet of about 2 mm thickness. The sheet was cut and pressed (2 mm) in a compression molding machine (Gotech, Qingdao, China), at 160 °C, for a time equal to the optimum cure time and 3.94 × 10^4^ kg/m^2^ ram dia pressure. While molding, Teflon^®^ sheets were placed between the sheet and the hot plates. The sheet was then cooled to room temperature by circulating cold water through the press plates. In AEGS, different amounts of K-SEP, 0 phr, 1 phr, 2 phr, and 3 phr were labelled AEGS0, AEGS1, AEGS2, and AEGS3, respectively. Similarly, in PEGS, different amounts of K-SEP, 0 phr, 1 phr, 2 phr, and 3 phr were labelled PEGS0, PEGS1, EPDM/PEGS2, and PEGS3, respectively. In this system, in order to verify the flame-retardant ability of sepiolite before and after modification, we added a group of samples AEGS1-M and PEGS1-M with only 1 phr of unmodified sepiolite as a control, respectively.

### 2.4. Measurements and Characterization

A Bruker Vertex 70 Fourier Transform Infrared Spectrometer (FTIR) was used to observe the infrared spectra of the silane coupling agent modified sepiolite; the ultimate oxygen index (LOI) of the two composites was tested using a HC-2 oxygen index meter from China Jiangning Analytical Instruments Co. Ltd. A CFZ-1 vertical combustion meter was used to test the vertical combustion grade (UL-94) of the two composites with a sample size of 130 mm × 13 mm × 3.2 mm, and the final grade was determined according to the standard described in ASTM D3801-2010 [30]. The ASTME 1354/ISO5660 international standard was used to measure the combustion behavior of the composites using a PX-07-007 cone calorimeter manufactured by Fire Test Technology, UK, with a sample size of 100 mm × 100 mm × 3 mm and a radiant heat flux of 35 kW/m^2^ [31]; a TGA-Q50 thermal analyzer from TA, USA, was used in a nitrogen (N_2_) atmosphere. The thermal weight loss (TGA) test was performed on the composite material under nitrogen (N_2_) atmosphere with a sample mass of 5 mg, the test temperature range was set from 30 °C to 800 °C, and the heating rate was 20 °C/min; the microscopic morphology of the combustion residue of the composite material was analyzed by means of a scanning electron microscope (SEM) of type JSM-6700FSEM-EDX from Japan Electronics Corporation with an accelerating voltage of 5 KV and a test magnification of ×10,000. The Raman spectroscopy of post-combustion residue of composite materials was performed using a high-resolution Bruker FRS-100S micro confocal Raman spectrometer with a CCD detector, spectral purity 700 cm^−1^~2100 cm^−1^, scanning wave number range 500–3000 cm^−1^; the mechanical properties (tensile strength and tear strength) of EPDM composite materials were analyzed using an AI-7000S general-purpose electronic tensile strength spectrometer. An AI-7000S universal electronic tensile testing machine was used to test the mechanical properties (tensile strength and tearing strength) of EPDM composites, the dumbbell-type specimens were prepared according to the ISO528-2009 standard with a tensile rate of 500 mm/min, and the cutting nozzle-type specimens were prepared according to the ISO528-2009 standard with a tearing rate of 200 mm/min [32].

## 3. Results and Discussion

### 3.1. Characterization of SEP and K-SEP

**Figure 2:**
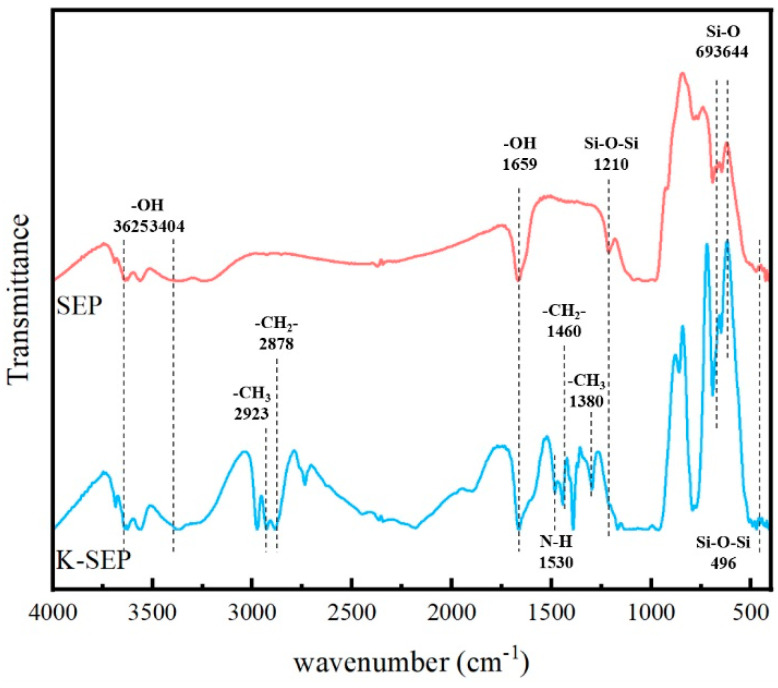
FTIR spectra of SEP and K-SEP.

To illustrate the modification of sepiolite (SEP) using the silane coupling agent KH550, spectroscopic analysis was conducted on the modified sepiolite (K-SEP) using FTIR as shown in Figure 2. The obtained spectrum of K-SEP was then compared with the spectrum of SEP.

The FTIR spectra of sepiolite reveal its magnesium–oxygen octahedral coordination structure, along with symmetric stretching vibration absorption bands at 3625 cm^−1^ and 3404 cm^−1^ corresponding to the -OH groups bound to water molecules. Additionally, the presence of zeolitic water in SEP is indicated by an in-plane bending vibration band of -OH at 1659 cm^−1^, while the bands at 1210 cm^−1^ correspond to the Si-O-Si structures within the silicon-oxygen tetrahedral structure [33]. Furthermore, an in-plane bending vibration band of the Si-O-Si structure is observed at 496 cm^−1^, while the Si-O stretching vibrational absorption bands associated with different O^2−^ structures in SEP appear at 693 cm^−1^ and 644 cm^−1^, respectively [25]. For K-SEP, new absorption bands emerge at 1460 cm^−1^ and 1380 cm^−1^, representing the symmetric stretching vibration and in-plane bending vibration of the methyl and methylene groups in KH550. Furthermore, absorption bands at 2923 cm^−1^ and 2878 cm^−1^ indicate the bending vibration of N-H, while the absorptions at both the O-Si and Si-O structures are increased, providing evidence for the successful modification of sepiolite by KH550. When KH550 modifies sepiolite, KH550 first reacts with water molecules on sepiolite, and the silicon ethoxy group is hydrolyzed to the silicon hydroxyl group, which forms hydrogen bonds with the hydroxyl group on sepiolite. When heated, a stable covalent connection is formed, so the absorption corresponding to the Si-O-Si structure and the Si-O structure is enhanced. After preparation, after repeated washing, the interference of free KH550 on the infrared band can be eliminated.

Figure S1 is the scanning electron microscope photos of AEGS2 and PEGS2. It can be seen from the picture that the sepiolite in AEGS2 has better dispersion and lower agglomeration than that in PEGS2. This may be due to the better binding ability of APP to sepiolite, which will show better flame-retardant and mechanical properties, which are verified by the flame-retardant and mechanical properties test below.

### 3.2. Flammability (UL-94 and LOI)

**Figure 3:**
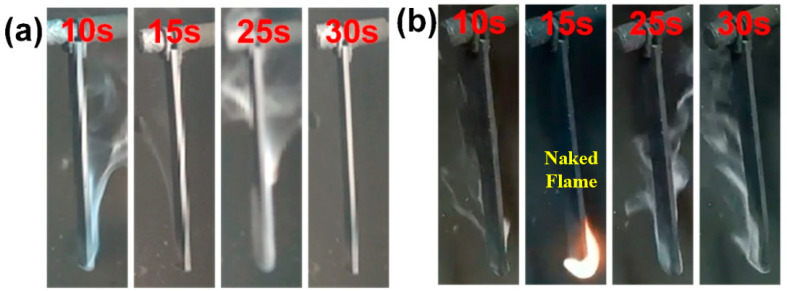
AEGS3 (**a**) and PEGS3 (**b**) vertical combustion process photos.

To evaluate the combustion performance of the two EPDM-based composites, tests were conducted to determine their ultimate oxygen index (LOI) and vertical combustion rating (UL-94). Table 1 presents the test results obtained for AEGS and PEGS, while Figure 3 displays photos capturing the vertical combustion process.

As presented in Table 1, the LOI values of the composites gradually increased with the addition of modified sepiolite, for both the composites with 21.1 phr EGT and 40 phr APP or PPAP. The LOI values of AEGS3 and PEGS3 composites increased by 8.1% and 5.5% compared to AEGS0 and PEGS0. Regarding the AEGS composites, they exhibited a UL-94 flammability rating of V-0, regardless of the presence or absence of modified sepiolite. Conversely, the PEGS composites achieved a UL-94 rating of V-0 only when 2 phr of modified sepiolite was added, with the flame being extinguished upon combustion source removal. As depicted in Figure 3a, the ignition process of AEGS composites showed no open flame, indicating the superior effectiveness of the AEGS intumescent flame-retardant system compared to the PEGS system. This may be due to the stronger binding ability of APP to sepiolite, and it can also be seen from the thermal decomposition curve that EPDM/AEGS composites decompose faster, while EPDM/PEGS decompose slower. Therefore, the LOI value of EPDM/AEGS is slightly higher than that of EPDM/PEGS. The addition of sepiolite significantly influences the combustion performance of EPDM-based composites by promoting the formation of a carbon layer, which acts as an insulation layer against heat and gas, ultimately enhancing the flame-retardant properties.

### 3.3. Cone Calorimetry Test

**Figure 4:**
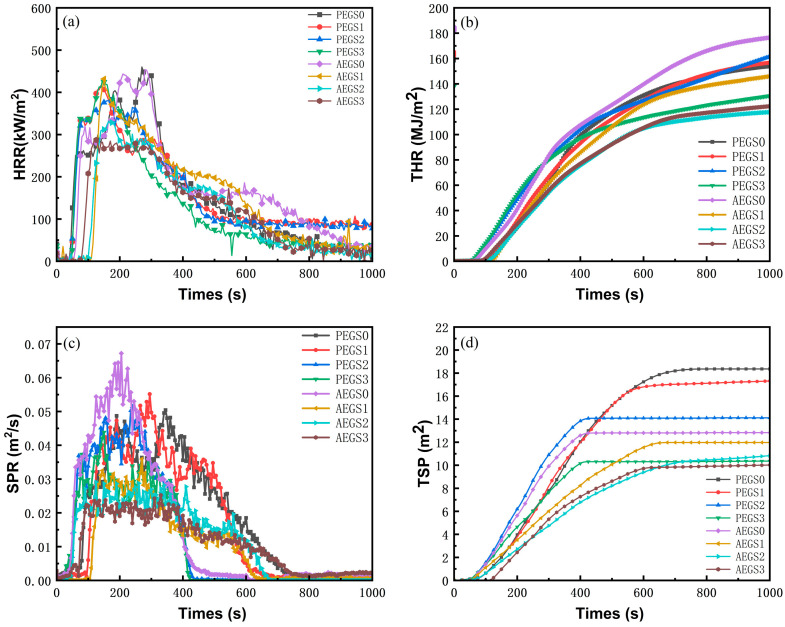
HRR (**a**), THR **(b**), SPR (**c**), and TSP (**d**) of EPDM/AEGS and PEGS composite materials.

**Figure 5:**
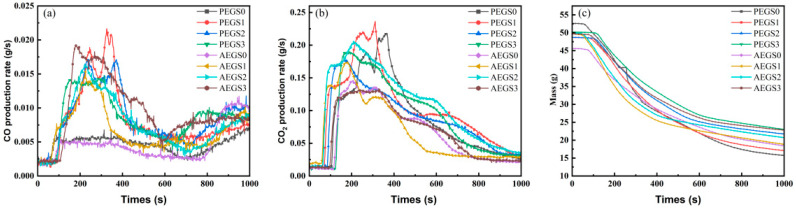
CO production rate (**a**), CO_2_ production rate (**b**) and Mass (**c**) of AEGS and PEGS composite materials.after CCT test.

To further examine the combustion properties of the two composites and understand the mechanism behind the promotion of carbon formation and smoke suppression properties resulting from the addition of modified sepiolite K-SEP, a conical calorimeter was employed to quantitatively assess the combustion properties of the composites; the relative data are shown in Figure 4 and Figure 5.

As depicted in Figure 4a, AEGS0 and PEGS0 without sepiolite exhibit relatively easy ignition. However, when sepiolite, a silicate clay-like mineral component, is incorporated into the rubber matrix, it adheres to the carbon layer during combustion. The internal channels and pores of sepiolite facilitate heat conduction and prevent heat accumulation, thereby reducing the material’s surface ignitability. Consequently, the addition of K-SEP leads to a delayed time to ignition (TTI). Upon the addition of 3 phr K-SEP, the band heat release rate (PHRR) of AEGS3 reached 284.4 kW/m^2^, while that of PEGS3 reached 410.8 kW/m^2^. Compared with the samples without sepiolite, they decreased by 37.1% and 10.7%, respectively. Compared with AEGS3, the PHRR decrease of the PEGS composites was slower. By observing the PHRR data of the PEGS composites, it can be seen that the PHRR of PEGS2 was higher than that of PEGS3. This may be due to the failure to achieve good interface compatibility between PPAP and sepiolite, resulting in a concentrated exothermic phenomenon caused by severe combustion at a certain node. By observing the FPI and FIGRA data, it can be seen that the fire risk of AEGS seems to be higher than that of PEGS composites, and it is speculated that there should be an optimal ratio between sepiolite and AEGS and/or PEGS.

Sepiolite demonstrates a positive influence on heat transfer acceleration and the formation of a char layer. Figure 5 shows the CO production rate, the CO_2_ production rate and the mass of AEGS and PEGS composite materials after combustion. As depicted in Figure 5c, the addition of K-SEP resulted in a 45.86% increase in residual char for PEGS3 compared to PEGS1, and a 23.91% increase for AEGS3 compared to AEGS1. Therefore, as the K-SEP content increases, it promotes the conversion of the composite into more char during combustion, establishing a physical barrier that slows down the combustion process. Consequently, the heat release rate of composites during combustion gradually diminishes. From Figure 4c,d, it is evident that K-SEP significantly enhances smoke suppression for the studied composites. The TSP values of AEGS3 and PEGS3 decreased by 20.3% and 42.39%, respectively, compared with AEGS0 and PEGS0. The incorporation of K-SEP, with its abundant internal channels and pore structure, effectively absorbs small particulate matter generated by incomplete combustion during polymer combustion. Furthermore, K-SEP promotes increased carbon formation in the composite material, reducing the extent of the combustion reaction and consequently lowering the production of particulate matter during combustion.

Figure 5a illustrates that during the initial stage of combustion, the incomplete combustion of the composite material occurs due to insufficient oxygen supply, resulting in the generation of a significant amount of carbon monoxide (CO) gas. However, as the combustion progresses, the high specific surface area and porous structure of sepiolite positively contribute to heat transfer and oxygen penetration, thereby slowing down the combustion process of the composite material. Sepiolite promotes the formation of carbon in the composite, leading to the production of carbon with higher specific gravity. Consequently, the amount of gas generated after complete combustion is reduced. As observed in Figure 5c, as the combustion time increases, the mass loss rate of the composites gradually decreases, resulting in a reduction in the smoke and gas produced during combustion. This transformation is attributed to the formation of an expanded carbon layer. Notably, the addition of K-SEP makes the residual carbon content of the PEGS composite system higher than that of the AEGS composite system. The observed residual carbon content aligns with the results obtained from the limiting oxygen index (LOI) test and the vertical combustion rating (UL-94) test conducted earlier. The addition of K-SEP serves as a catalyst for carbon formation in the cohesive phase mechanism of polymer flame retardancy and aids in reducing the generation of toxic and harmful gases, such as CO_2_ and CO, in the gas phase mechanism.

The ignition time (TTI) of the two composites is presented in Table 2, and TTI serves as a crucial indicator for evaluating the flame resistance of composites. A longer TTI signifies stronger flame resistance and a reduced fire hazard [26]. Upon analyzing the ignition time results, it is evident that the amount of sepiolite has a positive impact on the ignition time of the composites. Increasing the amount of sepiolite leads to an extended ignition time for both composites. To gain a better understanding of the fire risk associated with the composites, two indices were selected: the fire performance index (FPI) and the fire risk index (FIGRA). FPI represents the ratio of TTI to PHRR, providing insight into the occurrence of rapid ignition. A smaller FPI value indicates a shorter ignition time and higher fire risk. FIGRA, on the other hand, is defined as the ratio of PHRR to HRR band time, where a larger FIGRA value suggests a shorter time for the composite material to reach its band heat release rate and therefore a higher fire risk [34]. The FPI and FIGRA values for the two composites are listed in Table 2. The addition of sepiolite enhances the fire performance of the AEGS and PEGS composite systems. This improvement is likely due to the formation of a thin carbon layer at the onset of combustion, facilitated by the presence of sepiolite. The carbon layer acts as a barrier, minimizing the external heat’s erosion on the matrix and hindering the entry of oxygen. Consequently, the ignition time is delayed and the band heat release rate is reduced, thus enhancing the fire resistance of the material.

**Figure 6:**
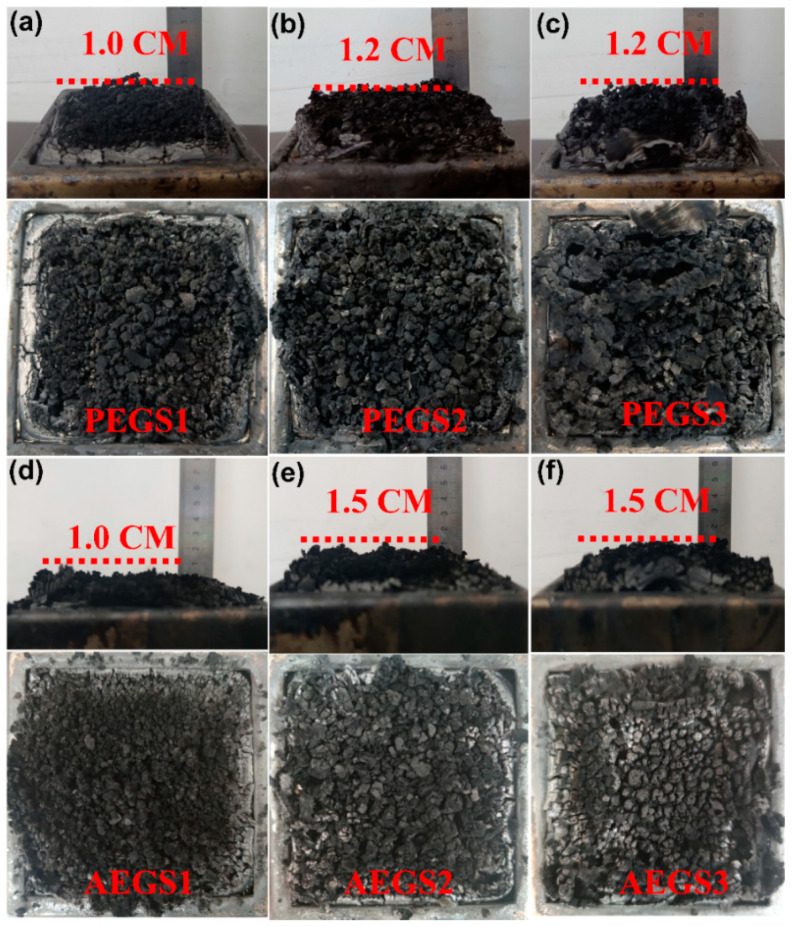
Digital photos of carbon residues of composite materials tested by cone calorimetry: (**a**) PEGS1, (**b**) PEGS2, (**c**) PEGS3, (**d**) AEGS1, (**e**) AEGS2, and (**f**) AEGS3.

Figure 6 displays the char residue morphology after combustion of both composites, revealing a prominent expansion of the char layer, particularly when 2 phr or even 3 phr of K-SEP is added. The presence of a phosphorus and nitrogen-based intumescent flame-retardant system in the composite leads to the thermal decomposition of APP and PPAP, resulting in the production of acids and gases that cause the matrix and graphene to expand and carbonize. This expansion acts as a barrier, isolating the heat and preventing gas penetration. The addition of K-SEP further reduces the heat release and smoke generation of the composite, resulting in a larger portion of the matrix transforming into carbon residue. Furthermore, K-SEP plays a crucial role in promoting the formation of a carbon layer, which becomes the framework connecting the carbon layer after material combustion. This stabilization of the swollen carbon layer leads to an increase in residual carbon height.

### 3.4. Thermal Stability

**Figure 7:**
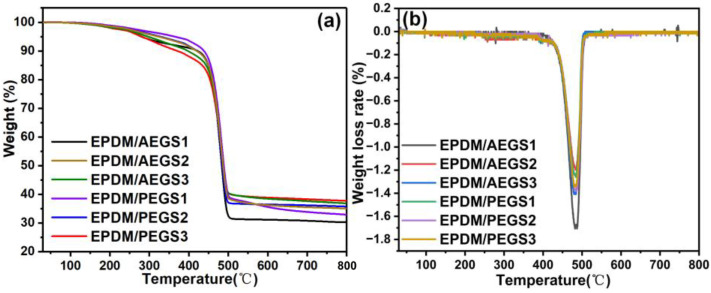
TG curve (**a**) and DTG curve (**b**) of EPDM/AEGS and EPDM/PEGS composites under the N_2_ atmosphere.

Thermal weight loss tests were conducted on the two composites under a N_2_ atmosphere to investigate their thermal stability. Figure 7 illustrates the TG curves (a) and DTG curves (b) of the composites, providing valuable insights into their thermal behavior in the N_2_ atmosphere.

As depicted in Figure 7a, the thermal weight loss of the two composites mainly occurs in the stage of 370~500 °C. In the initial stage of 250~370 °C range, the evaporation of bound water in sepiolite takes place, accompanied by a slight thermal decomposition in the phosphorus and nitrogen-based intumescent flame-retardant system within the composite. This decomposition generates NH_3_ and H_2_O, leading to weight loss, and initiates the formation of a small intumescent carbon layer in the composite, resulting in a faint DTG band and minimal mass loss. In the main thermal weight loss temperature range of 370~500 °C, the composites undergo charring, characterized by a prominent DTG band and a mass loss of nearly 50% [35]. The final residual carbon amount increased by 30.78% and 38.26 for the AEGS3 and PEGS3 composites, respectively, compared to the AEGS0 and PEGS0 composites. As shown in Figure 7b, K-SEP has a notable impact on the thermal decomposition of the composites within the temperature range of 370 °C to 500 °C. This influence is evident in the significant reduction of the maximum thermal decomposition rate, primarily attributed to sepiolite’s ability to promote char formation in the matrix, enhancing the strength of the char layer. As a result, substances that would otherwise be lost with heat are transformed into solid carbon, resulting in an increase in the mass of the final residue.

### 3.5. Microstructure Analysis

To further investigate the impact of K-SEP addition on the combustion of the composites, scanning electron microscopy (SEM) was employed to analyze both the surface and interior of the combustion residue in the K-SEP composites with the inclusion of 3 phr K-SEP, as illustrated in Figure S2.

Based on the information presented in Figure S2, the carbon residues on the surface of AEGS composites exhibit smaller size and greater completeness (Figure S2a,b) than that of PEGS composites (Figure S2c,d). Meanwhile, the presence of the phosphorus and nitrogen swelling flame-retardant APP leads to the generation of NH_3_ and H_2_O gases during combustion, resulting in some small pores within the carbon layer (Figure S2c). On the other hand, the carbon residues of PEGS composites are relatively larger in size and possess a smoother surface (Figure S2d,e). With the addition of K-SEP, a dense char layer forms after combustion due to its catalytic effect on char formation, effectively isolating the entry of heat and gas flow. This catalytic charring effect of K-SEP may be due to the reaction of Si-OH on the surface of K-SEP with APP or PPAP to form P-O-Si bonds, thus forming new phosphorus compounds (SiP_2_O_7_ and NH_4_Mg (PO_3_)_3_). It improves the thermal stability of APP or PPAP at high temperature, so that the phosphoric acid produced by the decomposition of APP or PPAP is more likely to catalyze the carbon source into carbon, and the free fibrous sepiolite plays a reinforcing role in the newly formed carbon layer, which can be used as part of the carbon layer skeleton [36]. Furthermore, the internal carbon residue of the composites in this system exhibits greater continuity after combustion compared to the others (Figure S2f). K-SEP synergistically interacts with GE, resulting in a more stable structure of the carbon residue formed during combustion. Additionally, GE also provides reinforcement for residue. From the SEM images of carbon slag, it can be seen that a stable and dense carbon layer is formed, sepiolite is used as a skeleton stable structure in the formed carbon layer, and a clear carbon slag phase structure can be observed. The graphene is used as a charring agent and become one of the components of the carbon slag, showing a continuous and dense structure. The use of K-SEP provides sufficient skeleton support for the carbon slag, making the carbon slag more stable. Moreover, K-SEP, with its inherent structural characteristics of numerous pores and a large internal surface area, exhibits enhanced adsorption capacity for the generated smoke. In summary, the K-SEP serves a triple function of catalysis, reinforcement, and smoke suppression, contributing to the formation of an effective flame-retardant carbon layer with smoke suppression capabilities.

### 3.6. Raman Spectroscopic Analysis

**Figure 8:**
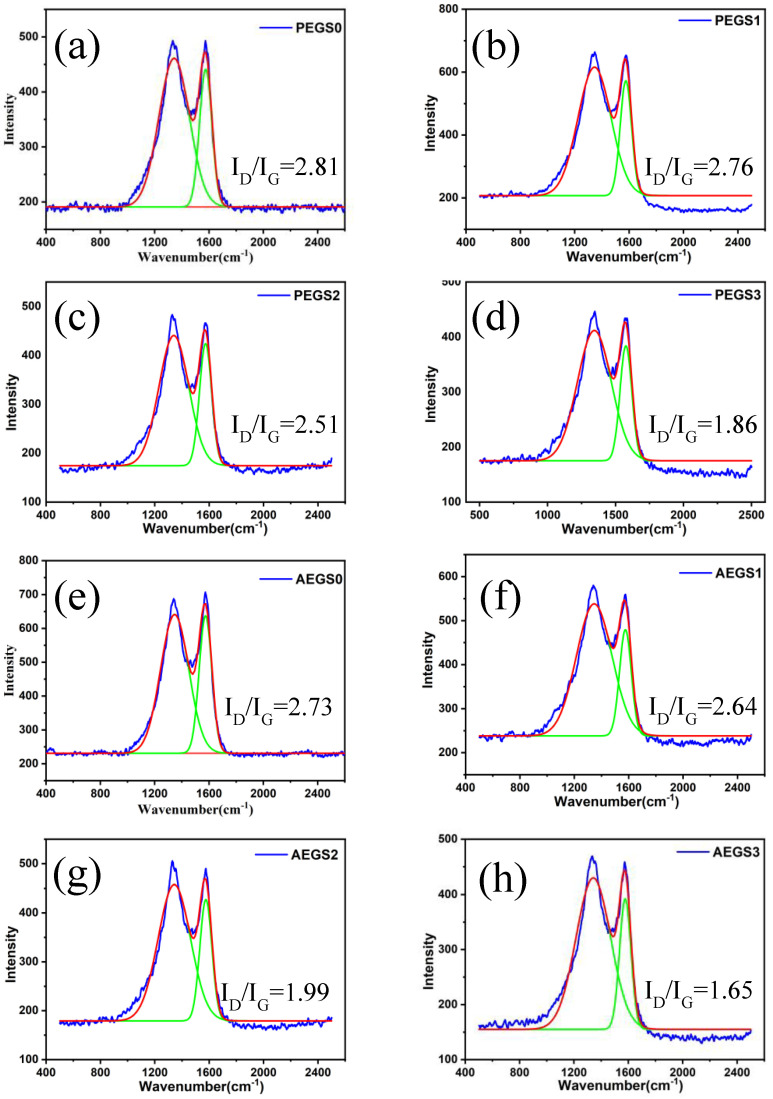
Raman spectra after band fitting of various EPDM composite carbon residues: (**a**) PEGS0, (**b**) PEGS1, (**c**) PEGS2, (**d**) PEGS3, (**e**) AEGS0, (**f**) AEGS1, (**g**) AEGS2, and (**h**) AEGS3.

To conduct a detailed investigation into the influence of K-SEP addition on the expanded char layer formed post-combustion, Raman spectroscopy was employed to analyze the char residue obtained from conical calorimetric testing. The corresponding results are presented in Figure 8.

The Raman spectra analysis of the carbon residue revealed the presence of the D-band and G-band at 1355 cm^−1^ and 1586 cm^−1^, respectively, indicating the presence of an amorphous carbon structure as shown in Figure 8. The D-band is associated with crystal defects in the C-atom structure, while the G-band corresponds to the in-plane stretching vibration of C-atom sp^2^ hybridization. Both bands are closely related to sp^2^ hybridization. To assess the structural order, the I_D_/I_G_ ratio, which represents the ratio of the areas under the D-band and G-band, is commonly used. A smaller I_D_/I_G_ value indicates a higher degree of structural order [37,38].

The I_D_/I_G_ ratio was employed to assess the degree of graphitization in the char residue. It was observed that the I_D_/I_G_ values decreased significantly with the increase in K-SEP content in both AEGS and PEGS composites. This finding further supports the role of K-SEP in promoting char formation and enhancing the graphitization of the char residue after combustion. A higher degree of graphitization in the char layer corresponds to improved shielding properties for solid-phase flame retardants and enhanced flame-retardant performance.

### 3.7. Mechanical Properties

It is widely acknowledged that the addition of fillers can reduce production costs and, in certain cases, enhance the physical and mechanical properties of rubber, thereby influencing their utilization conditions. However, as the quantity of nano-fillers increases, their large specific surface area and high surface energy tend to result in agglomeration within the rubber matrix, creating “defects” that often act as initiation sites for damage under stress conditions [39]. As shown in Table S1, the tensile strength of both composites displayed a trend of initially increasing and then decreasing as the amount of modified sepiolite increased. The optimal strength was achieved with AEGS2 and PEGS2, resulting in 11.09 MPa and 8.95 MPa, representing a 14.9% and 14.1% increase compared to AEGS0 and PEGS0. Conversely, the tear strength of AEGS and PEGS composites exhibited an increasing trend with the augmentation of K-SEP, showing improvements of 27.7% and 17.6% for AEGS3 and PEGS3 compared to AEGS0 and PEGS0. Therefore, the AEGS composites seem to be better in terms of mechanical properties than those of PEGS composites, which may be due to the reduction of mechanical properties caused by the dispersion of PPAP. Most of the flame retardants in the current research are developed at the expense of damaging the mechanical properties, and the amount of flame retardants required to achieve the required flame retardancy is high. The tensile strength data of the PPAP group seem to show that K-SEP has a weak reinforcing effect at a certain amount of addition. K-SEP possesses a high internal surface area due to its 2:1 lamellar structure, as well as numerous channels and pores containing zeolitic water and cations like potassium and calcium. When incorporated into the rubber matrix, K-SEP can adsorb a portion of the added nano-fillers within its channels and pores, facilitating its uniform dispersion throughout the rubber [22]. Consequently, the formation of “defects” arising from nano-filler aggregation is significantly reduced compared to systems without sepiolite addition. Moreover, the silicone hydroxyl groups present on the surface of KH550-modified sepiolite can interact with the polar polymer matrix, improving interfacial interactions and complementing the rubber matrix. The combined effects of these two aspects enhance the physical and mechanical properties of the composite. However, excessive amounts of nano-fillers diminish the adsorption effect of channels and pores, while excessive K-SEP addition can lead to poor dispersion due to its strong internal covalent bonding, resulting in degraded physical and mechanical properties.

## 4. Conclusions

Under the premise of not affecting the rubber processing process of EPDM, the two flame-retardant systems studied, AEGS and PEGS, show excellent flame-retardant and smoke suppression properties, especially in terms of mechanical properties; they also show the incomparable advantages of other traditional flame retardants, and have a certain strengthening effect on EPDM under a certain addition fraction of flame retardants. The main focus of this investigation was to evaluate the impact of the compounded AEGS and PEGS flame-retardant systems on the flame and smoke suppression performance of EPDM. The PHRR of AEGS3 and PEGS3 composites was 284.4 kW/m^2^ and 410.8 kW/m^2^, respectively, with a 37.1% decrease in PHRR compared to AEGS0 and PEGS0. Furthermore, the THR values of AEGS3 and PEGS3 composites decreased by 30.75% and 15.17%, and the TSP values decreased by 20.3% and 42.39. AEGS3 and PEGS3 composites led to a 21.3% and 30.4% increase in residual carbon content, demonstrating the positive impact of the porous structure of sepiolite on the flame-retardant and smoke suppression properties of EPDM. SEM and Raman spectroscopy analysis of the carbon residue of AEGS and PEGS composites after combustion revealed that the incorporation of sepiolite resulted in a more graphitized and denser expanded carbon layer, effectively isolating heat and gases. Additionally, the porous structure of sepiolite facilitated the rapid dissipation of internal heat and adsorption of solid micro-particles and gases generated during combustion. Therefore, the introduction of sepiolite significantly enhanced the catalytic carbonization efficiency of the studied AEGS and PEGS flame-retardant systems, and effectively inhibited the release of EPDM smoke.

## Figures and Tables

**Table 1:** LOI and UL-94 test data of EPDM matrix composites.

Sample	LOI ± 0.3 (%)	UL-94	Drip
AEGS0	31.2	V-0	NO
AEGS1-M	31.1	V-0	NO
AEGS1	31.5	V-0	NO
AEGS2	32.1	V-0	NO
AEGS3	33.7	V-0	NO
PEGS0	30.3	V-1	NO
PEGS1-M	30.3	V-1	NO
PEGS1	30.8	V-1	NO
PEGS2	31.2	V-0	NO
PEGS3	32.0	V-0	NO

**Table 2:** Cone calorimetry experimental data of various samples.

Sample	TTI	PHRR	THR	TSP	MASS	FPI	FIGRA	EHC
	s	kW/m^2^	MJ/m^2^	m^2^	g	10^−2^m^2^s/kW	kW/(m^2^/s)	MJ/kg
PEGS0	50	460.0	153.6	18.4	15.7	10.9	3.01	39.86
PEGS1	55	431.5	156.6	14.4	17.1	12.7	2.97	34.64
PEGS2	58	378.1	161.1	12.8	21.7	15.3	2.60	32.58
PEGS3	58	410.8	130.3	10.6	22.9	14.1	2.83	32.23
AEGS0	65	452.2	176.6	12.8	18.4	14	2.63	38.84
AEGS1	74	417.2	141.8	11.9	18.8	17.7	2.45	28.32
AEGS2	65	331.5	117.6	10.8	20.7	19.6	1.95	23.43
AEGS3	78	284.4	122.3	10.2	22.8	27.4	1.67	21.56

## Data Availability

The original contributions presented in the study are included in the article; further inquiries can be directed to the corresponding author/s.

## Supplementary Materials

The following supporting information can be downloaded at: https://www.mdpi.com/article/10.3390/polym16162281/s1, Figure S1: SEM images of the two composites. Among them, (a) and (b) are AEGS2 at ×3000 and ×30,000 magnifications, (c) and (d) are PEGS2 at ×3000 and ×30,000 magnifications. Figure S2: SEM photos of AEGS3 and PEGS3 composite materials: under 10,000 magnification (a) AEGS3 surface; under 30,000 magnification, AEGS3 surface (b), AEGS3 interior (c), 10,000 magnification PEGS3 surface (d); PEGS3 surface (e), PEGS3 interior (f) under 30,000 magnification. Table S1: Tensile strength and tear strength of EPDM matrix composites.
