# Supplementary material for: Enhancing Flame Retardancy and Smoke Suppression in EPDM Rubber Using Sepiolite-Based Systems
Source: Polymers (Basel). 2024 Aug 12;16(16):2281. doi: 10.3390/polym16162281 (PMC11359910; doi:10.3390/polym16162281)
Supplement: Supplementary file 1 [file polymers-16-02281-s001.zip › polymers-3142824-supplementary.pdf]

Supporting Information

Enhancing Flame Retardancy and Smoke Suppression in EPDM Rubber Using Sepiolite-Based Systems

Jiawang Zheng [1,†], Xu Zhang [1,†], Dawei Liu [2], Liwei Zhang [2], Yuxia Guo [2], Wei Liu [1], Shuai Zhao [1,*] and Lin Li [1,*]

[1] Key Lab of Rubber-Plastics, Ministry of Education/Shandong Provincial Key Lab of Rubber-Plastics, School of Polymer Science and Engineering, Qingdao University of Science and Technology, Qingdao 266042, China; zjw15164181831@163.com (J.Z.); sdbhjebdhehe@163.com (X.Z.); jksbkfbafhb@163.com (W.L.)

[2] Rike Chemical Co., Ltd., Weifang 262400, China; liudawei@rikechem.com (D.L.); zlw15698250119@163.com (L.Z.); guoyx1988@126.com (Y.G.)

* Correspondence: lyzhsh@163.com (S.Z.); qustlilin@163.com (L.L.)

† The authors contribute equally to this work.

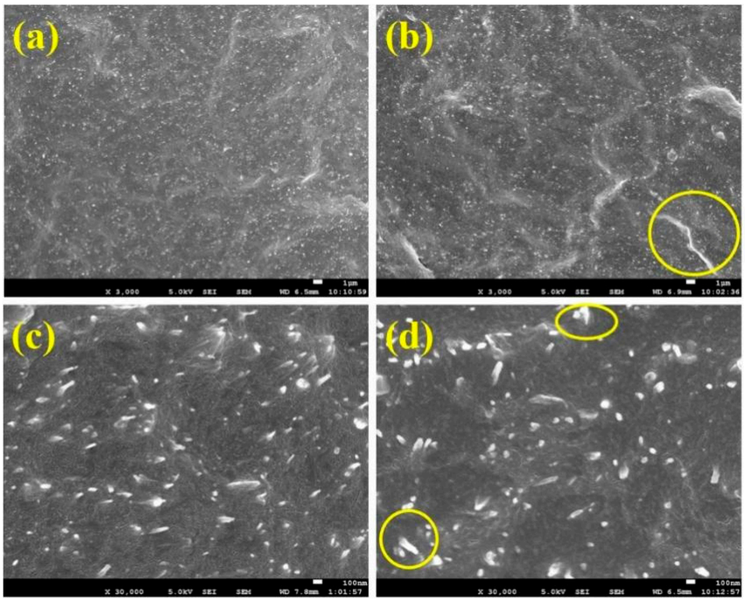

Figure S1. SEM images of the two composites. Among them, (a) and (b) are AEGS2 at × 3000 and × 30000 magnifications, (c) and (d) are PEGS2 at × 3000 and × 30000 magnifications.

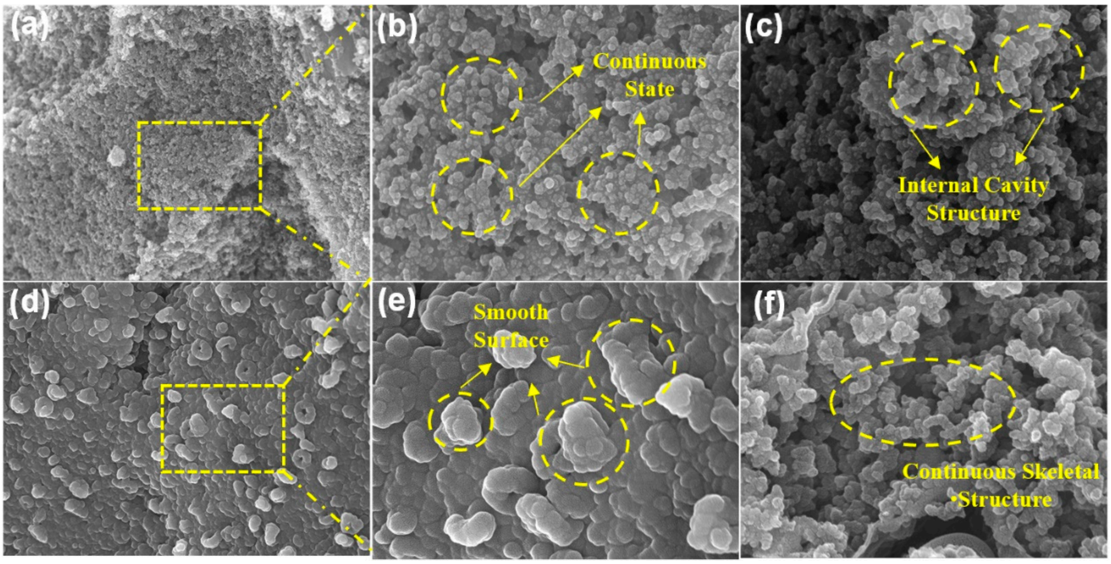

Figure S2. SEM photos of AEGS3 and PEGS3 composite materials: under 10000 magnification (a) AEGS3 surface; under 30000 magnification, AEGS3 surface (b), AEGS3 interior (c), 10000 magnification PEGS3 surface (d); PEGS3 surface (e),PEGS3 interior (f) under 30000 magnification

Table S1. Tensile strength and tear strength of EPDM matrix composites

Sample	Test Item				
	Tensile strength	Elongation	Modulus (100%)	Modulus (300%)	Tear strength
	MPa	%	MPa	MPa	N/mm
EPDM	8.62±0.05	282.01±0.13	2.82±0.02	8.6±0.25	27.64±0.33
AEGS0	10.40±0.12	309.87±0.36	2.89±0.27	9.95±0.12	43.64±0.014
AEGS1	10.15±0.015	298.73±0.27	3.07±0.36	9.64±0.40	48.73±0.16
AEGS2	11.09±0.10	318.27±0.34	3.08±0.28	10.39±0.39	52.60±0.42
AEGS3	9.96±0.036	292.04±0.29	2.99±0.14	7.48±0.18	55.75±0.18
PEGS0	7.85±0.04	287.65±0.28	2.24±0.05	—	27.64±0.46
PEGS1	8.30±0.12	320.17±0.17	2.01±0.09	6.67±0.27	28.50±0.34
PEGS2	8.95±0.29	328.93±0.29	2.19±0.07	6.77±0.19	29.82±0.28
PEGS3	8.45±0.15	298.54±0.16	2.36±0.16	8.18±0.014	32.53±0.19
